# Bio-Inspired CPG Modulation via Proprioceptive Deep Reinforcement Learning for Adaptive Hexapod Locomotion Across Terrain Transitions

**DOI:** 10.3390/biomimetics11080570

**Published:** 2026-08-09

**Authors:** Hao Jiang, Yuheng Lin, Zhihan Li, Liguo Shuai

**Affiliations:** 1 School of Mechanical Engineering, Southeast University, Nanjing 211189, China; 230208031@seu.edu.cn; 2 Jiangsu Collaborative Innovation Center of Atmospheric Environment and Equipment Technology, Nanjing University of Information Science & Technology, Nanjing 210044, China; 202612231060@nuist.edu.cn (Y.L.); lizhihan1994@163.com (Z.L.)

**Keywords:** hexapod robot, central pattern generator, deep reinforcement learning, proprioceptive control, terrain transition, adaptive locomotion, proximal policy optimization

## Abstract

Adaptive locomotion across continuous terrain transitions remains difficult for hexapod robots because contact timing, body attitude, support height, and load distribution change simultaneously along a route. This paper presents a unified proprioception-driven deep reinforcement learning and central pattern generator (DRL-CPG) framework for terrain-transition locomotion without visual terrain classification, explicit terrain labels, or terrain-specific controller switching. A high-level proximal policy optimization policy maps a 46-dimensional proprioceptive observation to a three-dimensional CPG modulation action comprising oscillation amplitude, swing-phase frequency, and turn modulation. A coupled six-node Hopf oscillator network then expands these modulated parameters into phase-coordinated rhythmic commands, which are mapped to the 18 joint targets of a JetHexa hexapod and executed by a low-level proportional-derivative controller. The observation space contains body linear velocity, body angular velocity, relative joint positions, relative joint velocities, the previous three-dimensional policy action, and inertial measurement unit (IMU)yaw/heading relative to the initial track direction. A continuous route consisting of flat ground, uphill stairs, irregular terrain, downhill stairs, and a recovery segment is defined to evaluate transition-aware locomotion using route completion, velocity-tracking error, lateral deviation, and roll/pitch fluctuation. Compared with the fixed-parameter CPG and end-to-end DRL baselines, the proposed method increased the full-distance success rate at 4.7 m from 9% and 20%, respectively, to 88%, while maintaining smoother velocity, lateral deviation, and roll/pitch responses. The framework preserves the rhythmic prior of CPG control while reducing the exploration burden of reinforcement learning, providing a compact formulation for adaptive hexapod locomotion across terrain transitions.

## 1. Introduction

Hexapod robots are well suited to field locomotion because distributed limb contacts enlarge the support polygon and tolerate local foot-ground uncertainty better than many wheeled platforms [1,2]. These features are valuable for inspection, search-and-rescue support, geological exploration, and hazardous-site operations, where deployment routes rarely remain homogeneous. Recent advances in intelligent sensing, visual–inertial localization, and learning-based component detection have further enhanced the perception and condition-monitoring capabilities of autonomous systems [3,4,5]. A robot may move from flat ground to stairs, traverse irregular surfaces, and descend within one mission. Such transitions perturb contact timing, load distribution, body attitude, velocity tracking, and lateral drift. Therefore, adaptive locomotion requires more than choosing a gait for a known terrain. The controller must regulate gait generation continuously from proprioceptive interaction states while preserving rhythmic continuity during terrain changes.

Hexapod locomotion controllers have mainly followed three directions: fixed or kinematic gait control, terrain-aware gait selection, and CPG-based rhythmic control. Fixed-gait approaches use predefined foot trajectories, inverse kinematics, stability criteria, and manually tuned timing rules, producing interpretable tripod, tetrapod, or wave gaits on regular terrain [6,7,8,9]. Terrain-aware variants improve adaptability by classifying terrain from vision, force-related features, or proprioceptive leg–terrain interactions [2,8,10,11]. Recent studies have further explored environment perception, contact-force optimization, and embodied adaptation to improve the terrain awareness and motion stability of robotic systems [12,13,14]. However, classification-based control depends on discrete labels that may be delayed or ambiguous in mixed transition regions. CPG-based control addresses rhythmic continuity by encoding leg coordination in coupled oscillator networks [15]. Recent hexapod studies have combined CPGs with reinforcement learning for adaptive gait generation, hierarchical control, and blind motion control [16,17,18,19,20], while broader legged-robot work has shown the value of coupling rhythmic priors with learned adaptation [21,22,23]. Yet CPG performance remains sensitive to amplitudes, frequencies, phase offsets, and trajectory mappings.

DRL provides a complementary adaptation mechanism because policies can learn from robot–environment interaction instead of relying entirely on hand-designed rules. More broadly, machine-learning methods have demonstrated considerable potential in forward prediction, inverse design, and intelligent dynamic modeling of complex engineering systems [24,25,26]. PPO is widely used in continuous-control problems because it balances update efficiency and implementation stability through a clipped surrogate objective [27]. Related developments in deep representation learning, intelligent optimization, and trustworthy sensing further demonstrate the broad applicability of data-driven methods to complex recognition and decision tasks [28,29,30]. In legged locomotion, DRL has enabled agile and robust skills over challenging terrain [31,32], with recent work emphasizing implicit terrain imagination, rapid adaptation, teacher–student learning, and continual reinforcement learning [33,34,35,36]. Related advances in learning-based locomotion have also emphasized robust policy transfer, sim-to-real optimization, and hybrid model- and data-driven control for legged robots [37,38]. For hexapods, directly learning residual commands for many joints increases the action dimension and can weaken gait interpretability. Proprioceptive DRL and CPG-assisted controllers partly address this issue [19,39,40], and sim-to-real or benchmarking studies clarify deployment requirements [41,42,43]. Related work on blind rough-terrain navigation and CPG-based control also supports structured adaptation [23,44]. Existing CPG-DRL hexapod studies demonstrate terrain-adaptive gait generation [16,17,18], but evaluations often emphasize isolated terrain classes rather than continuous transitions within a single route. Specialized expert-policy or gait-switching designs further adapt locomotion to terrain distributions [8,36,45], but switching among terrain-specific controllers can introduce discontinuities in timing, action distribution, and body response.

These limitations leave terrain-transition locomotion insufficiently resolved. Terrain-classification methods assume reliable discrete labels, specialized policy selection may create transient discontinuities, and high-dimensional joint-level actions increase exploration complexity for an 18-DoF hexapod [19,39,40]. Moreover, many evaluations report steady-state performance on single terrains, whereas post-transition recovery is less frequently quantified [2,7,44]. This paper therefore formulates adaptation as continuous CPG-parameter modulation rather than terrain-label recognition and controller switching. Proprioceptive observations describe the realized robot–terrain interaction, a CPG network maintains phase-coordinated rhythmic commands, and a learned policy adjusts a small set of rhythm parameters online. This formulation is consistent with recent hexapod CPG-DRL studies and broader CPG-RL locomotion research [21,22]. This study focuses on the transition-aware performance of a unified DRL-CPG controller.

**Figure 1:**
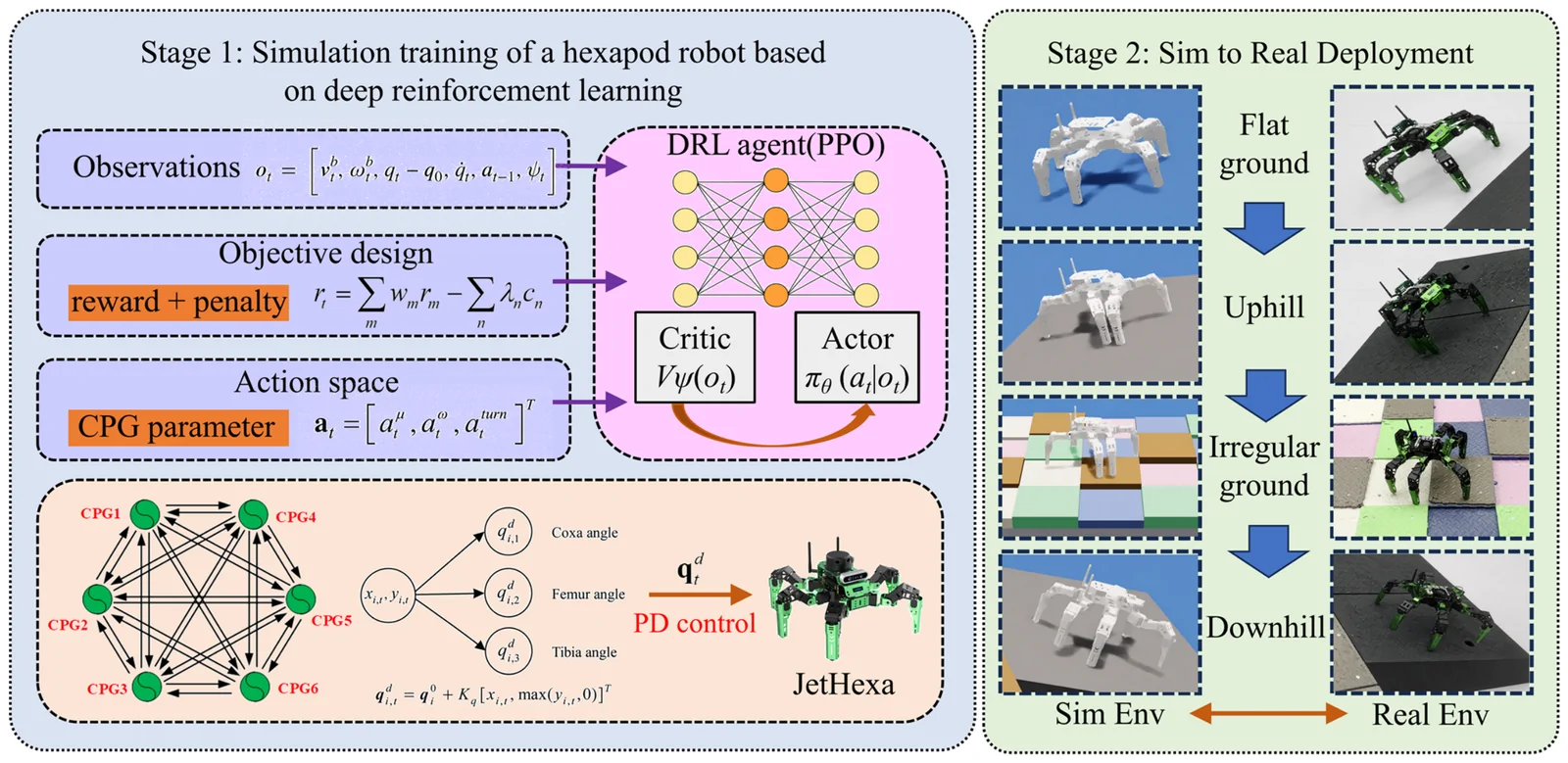
Technical route of the proposed unified proprioception-driven DRL-CPG framework. A single PPO policy maps proprioceptive states to low-dimensional CPG parameter increments; the coupled Hopf oscillator network expands these increments into rhythmic joint commands for the 18-DoF hexapod robot, and the learned controller is evaluated over continuous terrain transitions.

This paper proposes a unified proprioception-driven DRL-CPG framework for adaptive hexapod locomotion across continuous terrain transitions. The proposed controller uses one PPO policy to modulate selected CPG parameters from proprioceptive state information, rather than using a visual terrain classifier, discrete terrain label, or manually designed policy-switching mechanism. The technical route is summarized in Figure 1.

The main contributions of this paper are summarized as follows.

(1) A unified proprioception-driven DRL-CPG control architecture is developed for hexapod locomotion across continuous terrain transitions. The controller receives a 46-dimensional proprioceptive observation comprising body-frame velocities, relative joint states, the previous action, and relative IMU yaw/heading, and uses a single PPO policy to generate online CPG modulation commands. This design removes the need for explicit terrain classification or manual policy switching.
(2) A three-dimensional CPG parameter modulation mechanism is constructed to reduce the exploration burden of DRL while preserving the rhythmic structure of hexapod locomotion. Instead of outputting high-dimensional residual commands for all joints, the policy modulates oscillation amplitude, swing-phase frequency, and turn. The Hopf network expands these parameters into rhythmic commands for the 18 joints, while turn is implemented by differential scaling of the left and right Coxa targets.
(3) A continuous terrain-transition route is designed to evaluate locomotion beyond isolated single-terrain tests. The route contains flat ground, uphill stairs, irregular terrain, and downhill stairs within one sequence. The controller is assessed using training return, route success rate, velocity tracking, lateral deviation, and roll/pitch fluctuation, so that learning behavior, route completion, and locomotion stability during consecutive terrain changes can be quantified.

## 2. Preliminaries and Problem Description

This section defines the robot platform, rhythmic-control model, reinforcement-learning formulation, terrain-transition task, and PPO-based CPG parameter modulation method used in this study. The objective is to describe the control problem at a level that allows the controller to be reimplemented without relying on visual terrain labels or terrain-specific policy switching.

### 2.1. Hexapod Robot Platform

The JetHexa hexapod robot was used as the target platform. Each leg consists of three serially connected revolute joints, namely Coxa, Femur, and Tibia joints, resulting in 18 active degrees of freedom in total. The Coxa joint mainly drives the forward and backward swing of the leg in the horizontal plane, the Femur joint adjusts leg lifting and lowering, and the Tibia joint controls the extension and retraction of the distal segment. Similar kinematic modeling methods for multi-joint bionic robots have been applied in related research [46,47]. For the i-thleg, the joint-angle vector and the foot-end position in the local leg coordinate frame are defined as

(1) $$\left\{\begin{array}{l} q_{i}={\left[q_{i,1},q_{i,2},q_{i,3}\right]}^{T}\\ p_{i}={\left[p_{i,x},p_{i,y},p_{i,z}\right]}^{T} \end{array}\right.,\;\;i=1,2,\ldots ,6$$

Let the three link lengths be l_1, l_2, and l_3. The mapping from the single-leg joint space to the foot-end task space can be written as

(2) $$p_{i}=f_{i}\left(q_{i}\right)=\left[\begin{array}{c} \left[l_{1}+l_{2}\cos q_{i,2}+l_{3}\cos\left(q_{i,2}+q_{i,3}\right)\right]\cos q_{i,1}\\ \left[l_{1}+l_{2}\cos q_{i,2}+l_{3}\cos\left(q_{i,2}+q_{i,3}\right)\right]\sin q_{i,1}\\ -l_{2}\sin q_{i,2}-l_{3}\sin\left(q_{i,2}+q_{i,3}\right) \end{array}\right],\;\;i=1,2,\ldots ,6$$

The first two components describe how the Coxa joint changes the horizontal projection direction of the foot end, whereas the third component describes how the Femur and Tibia joints determine the vertical foot position. This kinematic relation specifies the mapping constraint between joint commands and foot motion and provides the basis for converting CPG rhythmic outputs into joint reference commands.

### 2.2. Coupled Hopf–CPG Network

To generate continuous and phase-coordinated rhythmic locomotion, a six-node Hopf oscillator network was used as the CPG. The state of the i-th oscillator is denoted as $z_{i,t}={\left[x_{i,t},y_{i,t}\right]}^{T}$, where $x_{i,t}$ and $y_{i,t}$ are two orthogonal oscillator components. Considering limit-cycle convergence, inter-leg phase coupling, and frequency switching between swing and stance phases, the coupled Hopf–CPG network is formulated as

(3) $$\left\{\begin{array}{l} {\dot{x}}_{i,t}=\alpha \left(\mu_{t}-{\left\|z_{i,t}\right\|}^{2}\right)x_{i,t}-\omega_{i,t}y_{i,t}+\sum_{j=1}^{6}\left(x_{j,t}\cos\phi_{ij}+y_{j,t}\sin\phi_{ij}\right),\\ {\dot{y}}_{i,t}=\alpha \left(\mu_{t}-{\left\|z_{i,t}\right\|}^{2}\right)y_{i,t}+\omega_{i,t}x_{i,t}+\sum_{j=1}^{6}\left(-x_{j,t}\sin\phi_{ij}+y_{j,t}\cos\phi_{ij}\right), \end{array}\right.\;i=1,2,\ldots ,6$$

Here, α is the limit-cycle convergence rate, $\mu_{t}$ is the target oscillation amplitude, $\omega_{i,t}$ is the instantaneous angular frequency of the i-th oscillator, and $\phi_{ij}$ is the desired phase difference between oscillators i and j. A tripod gait is used as the basic rhythmic pattern. The six legs are divided into two support groups, $\left\{L_{1},R_{2},L_{3}\right\}$ and $\left\{R_{1},L_{2},R_{3}\right\}$. Legs within the same support group remain in phase, whereas the two support groups maintain a phase difference of π.

To represent the temporal ratio between stance and swing phases and avoid abrupt frequency changes during phase transitions, a smooth frequency-switching mechanism is introduced. Let $\omega_{\mathrm{sw},t}$ and $\omega_{\mathrm{st},t}$ denote the swing-phase and stance-phase angular frequencies, respectively, and let β∈(0,1) denote the stance duty factor. The switching rule is

(4) $$\left\{\begin{array}{l} \omega_{\mathrm{st},t}=\frac{1-\beta }{\beta }\omega_{\mathrm{sw},t}\\ \omega_{i,t}=\left[1-\sigma \left(y_{i,t}\right)\right]\omega_{\mathrm{st},t}+\sigma \left(y_{i,t}\right)\omega_{\mathrm{sw},t}\\ \sigma \left(y_{i,t}\right)=\frac{1}{1+\exp\left(-by_{i,t}\right)} \end{array}\right.,\;\;i=1,2,\ldots ,6$$

The parameter b determines the transition steepness of the sigmoid function. This switching rule allows the CPG to move continuously between stance and swing phases, thereby reducing discontinuities in the target joint angles caused by abrupt frequency changes.

The CPG oscillator outputs are continuous two-dimensional rhythmic signals and must be further mapped to the target joint angles of the hexapod. A hierarchical joint-mapping strategy is used. The component $x_{i,t}$ generates the forward–backward swing of the Coxa joint and therefore regulates stride length, whereas $y_{i,t}$ generates the leg-lifting signal during swing. To avoid unnecessary foot lifting during stance, positive half-wave rectification is applied to $y_{i,t}$ and only its positive component is used as the lifting input for the Femur and Tibia joints. The target joint angle of the i-th leg is defined as

(5) $$q_{i,t}^{d}=q_{i}^{0}+\left[\begin{array}{cc} k_{c} & 0\\ 0 & k_{f}\\ 0 & -k_{t} \end{array}\right]\left[\begin{array}{c} x_{i,t}\\ \max\left(y_{i,t},0\right) \end{array}\right],\;\;i=1,2,\ldots ,6$$

In this expression, $q_{i}^{0}$ is the joint-bias vector in the nominal standing posture. The gains *k_c_*, *k_f_* and *k_t_* correspond to the Coxa, Femur, and Tibia mappings, respectively, and form the joint-mapping gain matrix **K***_q_*.

### 2.3. Reinforcement Learning Problem Formulation

Adaptive locomotion control of the hexapod robot across terrain transitions is formulated as a Markov decision process. At each discrete control instant, the robot selects an action according to its current proprioceptive state and receives an immediate reward through interaction with the simulated environment. The objective of reinforcement learning is to learn an optimal policy that allows the robot to maintain stable locomotion using only its own motion state, without visual terrain classification, explicit terrain labels, or specialized controller switching. This process is represented as

(6) $$M=\left(S,A,P,R,\gamma \right),\;\;J(\theta )=E_{\pi_{\theta }}\left[\sum_{t=0}^{T-1}\gamma^{t}r_{t}\right]$$

In the left expression, S,A,P,R,γ denote the state space, action space, transition function, reward function, and discount factor, respectively. The policy network is parameterized by θ, J(θ) is the expected accumulated discounted return, and $r_{t}$ is the immediate reward at time t. Unlike approaches that rely on visual terrain classification or explicit terrain labels, the proposed policy uses purely proprioceptive observations. The 46-dimensional observation consists of body-frame linear velocity, body-frame angular velocity, relative joint positions, relative joint velocities, the previous three-dimensional policy action, and relative IMU yaw/heading.

The action space is not defined as 18-dimensional joint targets or torques, but is restricted to low-dimensional CPG parameters. The PPO policy outputs a three-dimensional continuous action that modulates CPG amplitude, swing-phase angular frequency, and turn. The CPG network and Coxa differential mapping then generate 18-dimensional joint targets under phase-coordination constraints. The policy action is abstracted as

(7) $$a_{t}={\left[a_{t}^{\mu },a_{t}^{\omega },a_{t}^{turn}\right]}^{T}$$

The component $a_{t}^{\mu }$ adjusts the CPG amplitude and therefore affects stride length and leg-lifting height, $a_{t}^{\omega }$ adjusts the swing-phase angular frequency and therefore affects stride frequency and forward rhythm, and $a_{t}^{turn}$ represents the output steering modulation action.

### 2.4. Terrain-Transition Task Definition

This study targets adaptive hexapod locomotion over continuously changing terrain. A representative terrain-transition route is constructed from flat ground, uphill stairs, irregular terrain, downhill stairs, and a flat recovery segment to simulate slope changes, abrupt support-height variation, and contact uncertainty in field environments. During the entire task, the robot does not receive terrain-class labels, switch controllers, or reset the policy. It maintains a basic tripod rhythm and adjusts the CPG amplitude, swing-phase frequency, and turn coefficient online using proprioceptive feedback only. The transition-aware locomotion performance is evaluated in terms of whole-route traversal capability, velocity-tracking error, lateral deviation, and roll/pitch attitude fluctuation.

## 3. Methodology

The proposed method is a proprioception-driven DRL-CPG parameter modulation framework for terrain-transition locomotion. A high-level PPO policy generates CPG modulation actions online from proprioceptive observations. The CPG module then produces phase-coordinated joint targets, and a low-level PD controller executes the joint commands. Through this hierarchical structure, the policy only needs to learn adaptive modulation rules for gait parameters rather than constructing multi-joint periodic coordination from scratch.

### 3.1. Overall Control Framework

The control framework consists of four components: proprioceptive observation, PPO-based parameter modulation, CPG parameterized action mapping, and low-level joint control. In each control cycle, the environment returns a 46-dimensional observation including body linear and angular velocities, relative joint positions and velocities, the previous action, and relative IMU yaw/heading. The PPO policy maps this observation to a three-dimensional action. After bounding and parameter mapping, the action gives CPG amplitude, swing-phase frequency, and turn. The CPG generates phase-coordinated joint targets, turn differentially scales the left and right Coxa targets, and the PD controller computes joint torques from the target and measured states.

The central idea of this framework is to transform the high-dimensional joint-action search problem in terrain-transition locomotion into a three-dimensional gait-parameter modulation problem. The CPG provides a stable rhythmic prior, whereas PPO adjusts gait amplitude, swing-phase frequency, and steering online according to proprioceptive feedback. This design is intended to improve locomotion adaptability and stability during uphill stair climbing, irregular-terrain traversal, downhill stair descent, and recovery on flat ground.

### 3.2. Proprioceptive Observation Space

To improve the generalization capability of the controller in unknown terrain and terrain-transition processes, a purely proprioceptive observation is used as the policy input. According to Equation (8), the 46-dimensional observation consists of body linear and angular velocities, relative joint positions and velocities, the previous action, and relative IMU yaw/heading:

(8) $$o_{t}=\left[v_{t}^{b}\;\omega_{t}^{b}\;q_{t}-q_{0}\;{\dot{q}}_{t}\;a_{t-1}\;\psi_{t}\right]\in {\mathbb{R}}^{46}$$

Here, $v_{t}^{b}$ and $\omega_{t}^{b}$ are the body-frame linear and angular velocities, $q_{t}\in {\mathbb{R}}^{18}$ and ${\dot{q}}_{t}\in {\mathbb{R}}^{18}$ denote the joint angles and joint velocities, $q_{0}$ is the default joint configuration obtained by stacking the nominal joint-bias vectors $q_{i}^{0}$ of all legs, $a_{t-1}$ is the policy action from the previous time step, and $\psi_{t}$ is the IMU yaw/heading relative to the initial track direction. The component dimensions are 3 + 3 + 18 + 18 + 3 + 1 = 46. The previous action helps suppress abrupt variation between control cycles, while the heading term supports online steering correction. No explicit observation noise was injected during simulation training. During physical deployment, angular velocity was obtained from the filtered IMU signal, heading was derived by integrating the yaw rate, and the joint-state terms were constructed from the CPG reference commands and their finite-difference velocities rather than from servo-encoder measurements.

### 3.3. CPG Parameter Modulation Action Space

Unlike residual correction of CPG-generated joint angles, the PPO policy does not directly output joint-angle compensation. Instead, it produces three low-dimensional CPG modulation components from the current proprioceptive state. These components adjust gait amplitude, swing frequency, and turn while preserving phase coordination. Before entering the CPG module, the action is projected into a bounded feasible range and converted into actual CPG parameters:

(9) $${\hat{a}}_{t}=\Pi_{A}\left(a_{t}\right),\;\left[\begin{array}{c} \mu_{t}\\ \begin{array}{c} \omega_{\mathrm{sw},t}\\ \delta_{t} \end{array} \end{array}\right]=\left[\begin{array}{c} 0.5\\ \begin{array}{c} 4\pi \\ 0 \end{array} \end{array}\right]+\left[\begin{array}{ccc} 1 & 0 & 0\\ 0 & 8\pi & 0\\ 0 & 0 & 1 \end{array}\right]{\hat{a}}_{t}$$

The operator $\Pi_{A}$ denotes the action projection, and ${\hat{a}}_{t}$ is the projected action. Equation (9) maps it to $\mu_{t}$ (CPG amplitude), $\omega_{\mathrm{sw},t}$ (swing-phase angular frequency), and $\delta_{t}$ (turn coefficient). The third component sets the turn coefficient $\delta_{t}$, which scales the left and right Coxa targets by $1+\delta_{t}$ and $1-\delta_{t}$. The first two action components are bounded within [−0.3, 0.3], while the turn component and $\delta_{t}$ are bounded within [−0.25, 0.25]. These bounds were empirically selected around a stable open-loop CPG operating point to allow effective gait modulation while avoiding excessive joint targets and left–right asymmetry. This bounded mapping reduces abrupt parameter changes, and the mapped parameters are then used to generate the 18-dimensional target joint vector:

(10) $$q_{t}^{d}=f_{\mathrm{CPG}}\left(\mu_{t},\omega_{\mathrm{sw},t},\delta_{t},\Phi ,\beta \right)$$

Here, Φ is the desired inter-leg phase-relation matrix. Equation (10) combines phase coordination, CPG parameter modulation, and joint-target generation. The turn coefficient $\delta_{t}$ is applied by scaling the left and right Coxa targets by (1 + $\delta_{t}$) and (1 − $\delta_{t}$), respectively. Thus, the policy regulates three CPG-related parameters instead of directly searching the 18-dimensional joint space. Contact and force signals were excluded because they were unavailable on the physical robot; terrain interaction was inferred indirectly from IMU and joint-state information.

### 3.4. Reward and Constraint Design

To guide the hexapod robot toward stable, efficient, and transferable locomotion during terrain transitions, the reward function combines task rewards and constraint penalties. Task rewards encourage the robot to track the desired velocity, maintain heading consistency, and complete the route. Constraint penalties suppress vertical bouncing, body-attitude oscillation, excessive joint torque, lateral deviation, and failure termination. The total reward at each control cycle is defined as

(11) $$r_{t}=\sum_{m=1}^{M}w_{m}r_{m},t+\sum_{n=1}^{N}\lambda_{n}c_{n},t\;\;w_{m}\;>0,\;\lambda_{n}\;<0$$

The terms $r_{m}$ represent task rewards, including horizontal-velocity tracking, yaw angular velocity tracking, and goal-reaching rewards. The terms $c_{n}$ represent constraint penalties, including vertical velocity, roll/pitch angular velocity, joint torque, termination, lateral deviation, and out-of-bounds penalties. The coefficients $w_{m}$ and $\lambda_{n}$ are the corresponding reward and penalty weights. Their values are listed in Table 1. This formulation allows different locomotion objectives and constraints to be adjusted independently while keeping the reward structure compact and interpretable.

In Table 1, $v_{xy,t}^{\mathrm{cmd}}$ and $v_{xy,t}$ are the desired and actual horizontal velocities, respectively; $\omega_{z}^{\mathrm{cmd}}$ and $\omega_{z}$ are the desired and actual yaw angular velocities; $\sigma_{v}$ and $\sigma_{\omega }$ are scale factors in the velocity-tracking rewards. The variable $d_{x}$ denotes the longitudinal displacement of the robot relative to the initial position, $d_{y}$ denotes the lateral deviation relative to the track centerline, and $x_{\mathrm{off}}$ is the target longitudinal-distance threshold. I is an indicator function that equals 1 when the condition in parentheses is satisfied and 0 otherwise. The variable ρ is the normalized lateral deviation. The smoothness term *c_s_* aggregates three regularization terms, where $v_{z}$ $\omega_{xy}$, and τ denote the body vertical velocity, roll/pitch angular velocity vector, and joint torque vector, respectively. All translational velocities and distances are expressed in SI units of m·s^−1^ and m, respectively. Angular velocities are expressed in rad·s^−1^, relative yaw is expressed in rad, and joint torques are expressed in N·m. Accordingly, $\sigma_{v}$ and $\sigma_{\omega }$ have units of m·s^−1^ and rad·s^−1^, respectively, so that the arguments of the exponential reward functions are dimensionless.

### 3.5. PPO-Based Policy Optimization

The high-level parameter modulation policy is trained using proximal policy optimization. The Actor network takes the proprioceptive observation as input and outputs a distribution over a three-dimensional continuous action. The Critic network estimates the state value from the same observation and is used to compute the advantage estimate for policy updating. By constraining the magnitude of each policy update, PPO reduces the risk of performance degradation caused by overly large parameter updates and improves training stability in complex terrain-transition tasks. The training hyperparameters are listed in Table 2. A small learning rate is used to promote stable convergence of the Actor–Critic networks; the discount factor and generalized advantage estimation coefficient balance bias and variance in long-horizon return estimation; the clipping range limits the policy update magnitude; and multiple epochs with minibatch sampling improve sample utilization. Both the Actor and Critic use multilayer perceptron architectures to balance policy expressiveness and inference efficiency.

During each training cycle, the environment returns the current proprioceptive observation $o_{t}$. The Actor network samples a three-dimensional action $a_{t}$ from this observation. After clipping and parameter mapping, the action yields the CPG amplitude $\mu_{t}$ and swing-phase angular frequency $\omega_{\mathrm{sw},t}$, together with the turn coefficient *δ_t_*. The CPG module and left-right Coxa differential mapping then generate the 18-dimensional target joint vector $q_{t}^{d}$. The low-level PD controller computes joint torques from the target joint angles and measured joint states and applies them to the robot. The environment computes the reward according to velocity tracking, goal reaching, attitude stability, path maintenance, and actuation regularization, and then returns the next observation. The sampled trajectories are used to compute the advantage function, policy loss, and value-function loss, completing the PPO policy update. Through this optimization process, the policy learns the mapping from proprioceptive observations to CPG parameter actions within a unified control framework.

## 4. Experiments and Results

Simulation training and terrain-transition tests were conducted to evaluate the proposed unified proprioception-driven DRL-CPG parameter modulation method. The primary objective was to determine whether the robot could regulate rhythmic parameters online using proprioceptive information alone while traversing a single route composed of flat ground, uphill stairs, irregular terrain, downhill stairs, and a flat recovery segment. No visual terrain classifier, explicit terrain label, controller switching, or controller reinitialization was used. To assess the effectiveness of the proposed method, it was compared with a fixed-parameter CPG controller and an end-to-end DRL policy.

### 4.1. Experimental Setup

The simulation experiments were performed in Isaac Lab. The robot model adopted the JetHexa hexapod configuration and retained the main dynamic properties required for physically meaningful training, including body mass, link inertia, joint limits, and foot–ground contact. The test route consisted of flat ground, uphill stairs, irregular raised terrain, downhill stairs, and a flat recovery segment, which together evaluated locomotion adaptation under slope variation, abrupt support-height changes, and consecutive terrain transitions.

Training was conducted with the proposed DRL-CPG parameter modulation framework. The high-level PPO policy mapped a 46-dimensional proprioceptive observation to a three-dimensional CPG action. The observation included body linear and angular velocities, relative joint positions and velocities, the previous action, and relative IMU yaw/heading. The Hopf–CPG network generated phase-coordinated joint targets from the amplitude and frequency parameters, while the turn parameter differentially scaled the left and right Coxa targets before PD control. No terrain labels or terrain-specific controller switching were used during training or testing. During training, small initial joint-position and joint-velocity offsets were sampled from [−0.01, 0.01], and intermittent base-velocity perturbations of [−0.1, 0.1] m/s in the horizontal directions were applied every 10–15 s. The commanded forward velocity varied between 0.3 and 0.5 m/s. The ground, ramp, and irregular blocks used different but fixed contact materials; foot static and dynamic friction coefficients were both set to 1.0, the nominal robot mass was retained, the effective joint torque was limited to ±30 N·m, and no artificial observation or action delay was introduced. Therefore, the training employed initial-state and external-perturbation augmentation rather than comprehensive domain randomization.

The main evaluation metrics were episodic return, forward velocity, lateral deviation, roll and pitch attitude angles, and full-route traversal ability. Forward velocity quantified propulsion continuity, lateral deviation measured path-keeping performance, roll and pitch reflected body stability, and full-route traversal ability provided an overall assessment of task completion under continuous terrain perturbations. All subsequent comparisons among the proposed method, fixed CPG, and end-to-end DRL were conducted under matched route and evaluation conditions.

### 4.2. Training Convergence

**Figure 2:**
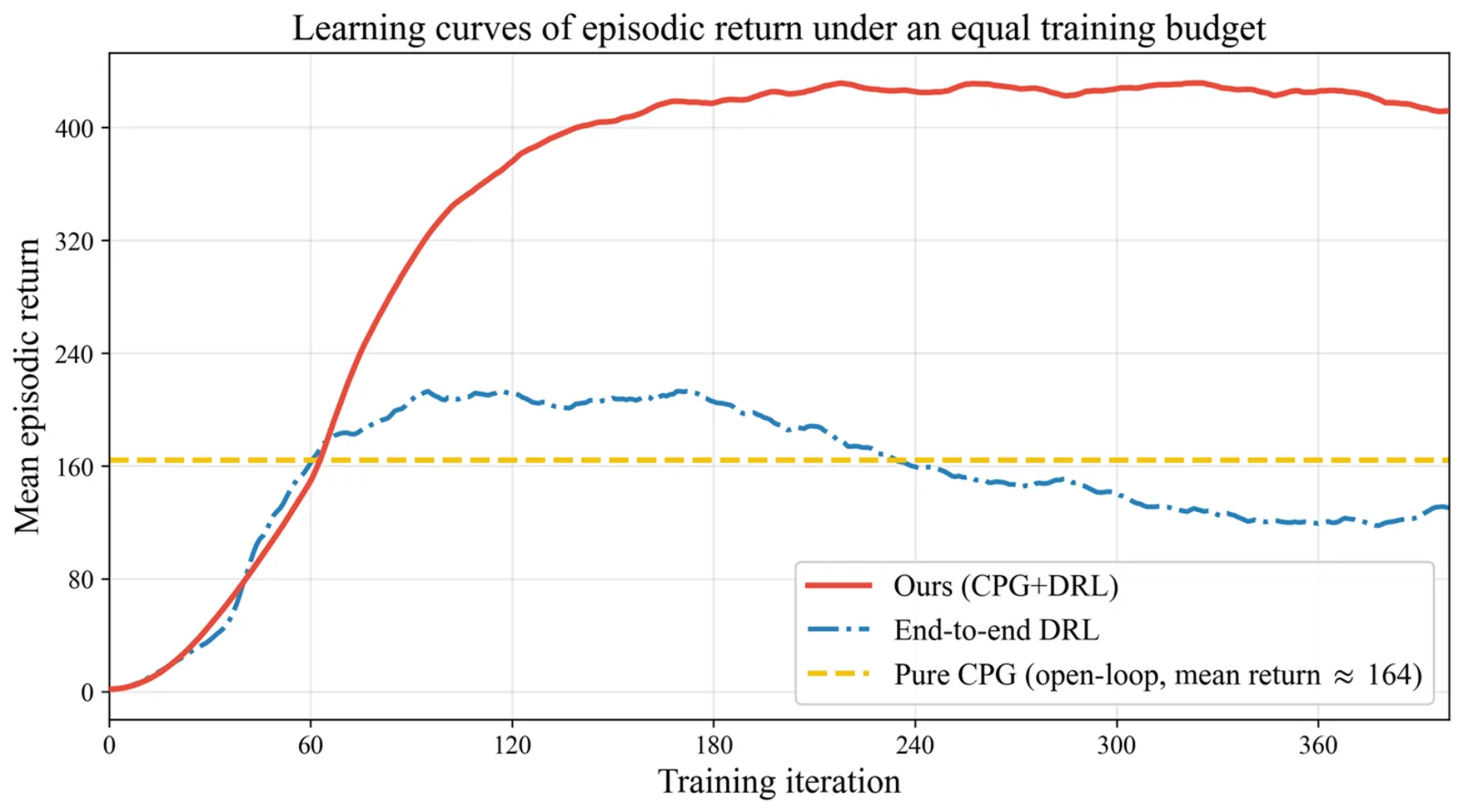
Learning curves of mean episodic return under an equal training budget.

To evaluate whether low-dimensional CPG-parameter modulation improved policy learning before route-level testing, the mean episodic returns of the three controllers were compared in Figure 2. The fixed-parameter Pure CPG controller did not participate in policy optimization, and its return remained at the open-loop reference level of approximately 198. The end-to-end DRL return initially increased and briefly reached or slightly exceeded the Pure CPG level around the middle of training, but it subsequently declined and remained mainly around 120–140 near the end. Direct policy learning in the 18-dimensional joint-action space therefore imposed a large search burden, because joint coordination, periodic rhythm generation, and terrain adaptation had to be explored simultaneously.

By contrast, the proposed CPG + DRL method showed higher sample efficiency and final performance. Its mean episodic return exceeded the Pure CPG reference after about 65–70 iterations, rose above 400 at about 140–150 iterations, and remained mainly around 420–430 in the later stage. Restricting the action to three CPG parameters reduced the search burden, allowing the policy to learn amplitude, swing-phase frequency, and turn modulation on top of the CPG rhythmic prior.

Table 3 reports the success rates of the three methods at key forward distances along the continuous terrain route. At 1.3 m and 1.6 m, the proposed method maintained 100% success, whereas Pure CPG achieved 59% and 54% and end-to-end DRL achieved 95% and 91%, respectively. As the traveled distance increased, the success rates of both baselines declined rapidly. Pure CPG decreased to 22%, 17%, 11%, and 9% at 3.1 m, 3.4 m, 4.2 m, and 4.7 m, respectively. End-to-end DRL achieved 36%, 32%, 22%, and 20% at the same distances, showing that early-stage motion did not translate into reliable full-route traversal.

The proposed method maintained more stable task completion at longer distances. At 2.8 m, 3.1 m, 3.4 m, 4.2 m, and 4.7 m, the CPG + DRL success rates were 96%, 94%, 93%, 91%, and 88%, respectively, which were consistently higher than those of the two baselines. These results show that the CPG rhythmic prior helped preserve basic gait coordination, while DRL-based three-parameter modulation adjusted stride, rhythm, and steering online from proprioceptive feedback, thereby improving robustness over consecutive terrain disturbances.

Taken together, Figure 2 and Table 3 show that the proposed method improved both training convergence and long-distance route completion. Compared with fixed CPG and end-to-end DRL, CPG + DRL achieved a more favorable balance among rhythmic stability, online adaptability, and sample efficiency for hexapod locomotion over continuous complex terrain.

To examine the contribution of the three CPG modulation channels, controlled ablation experiments were conducted under the same terrain route, training seed, training budget, and 1400-iteration evaluation checkpoint, as shown in Table 4. The complete amplitude–frequency–turn policy achieved an 87.89% success rate, whereas removing turn modulation, retaining amplitude only, and retaining frequency only reduced the success rate to 12.89%, 3.52%, and 0.00%, respectively, indicating that amplitude and frequency provide complementary stride and timing adaptation, while turn modulation is essential for suppressing accumulated lateral drift.

### 4.3. Simulation Results and Quantitative Analysis

**Figure 3:**
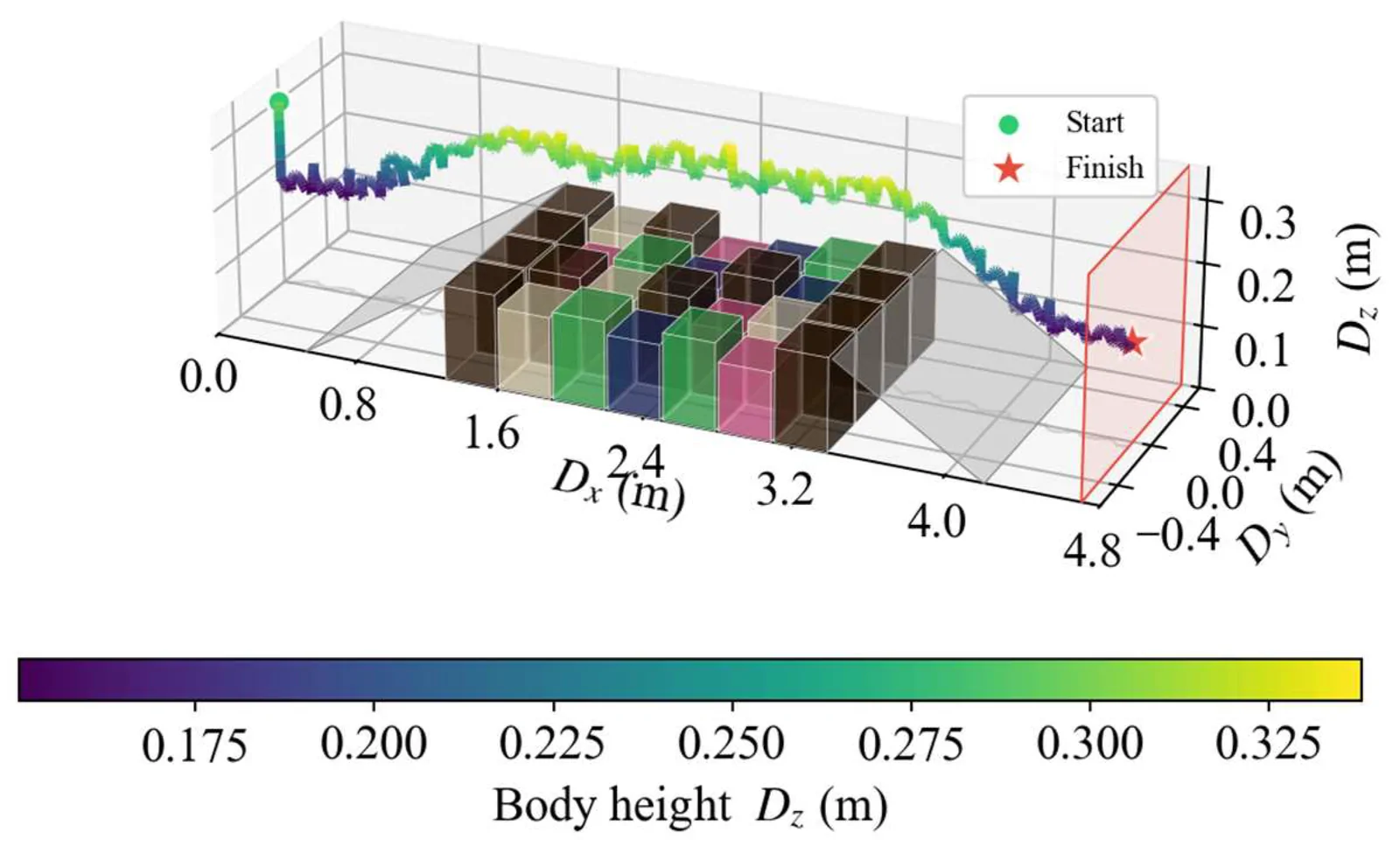
Three-dimensional trajectory of the proposed CPG + DRL method over the compound terrain. The trajectory color denotes body height.

To connect the convergence and success-rate results with actual terrain traversal, the trajectory of the proposed method over the compound route is visualized in Figure 3. The robot traversed the irregular raised region and reached the finish area. During traversal, the trajectory color represents the measured body height. The body height increased as the robot entered the raised terrain and decreased after it left the obstacle region, indicating that the controller adjusted the CPG parameters online in response to changes in support height and body state.

**Figure 4:**
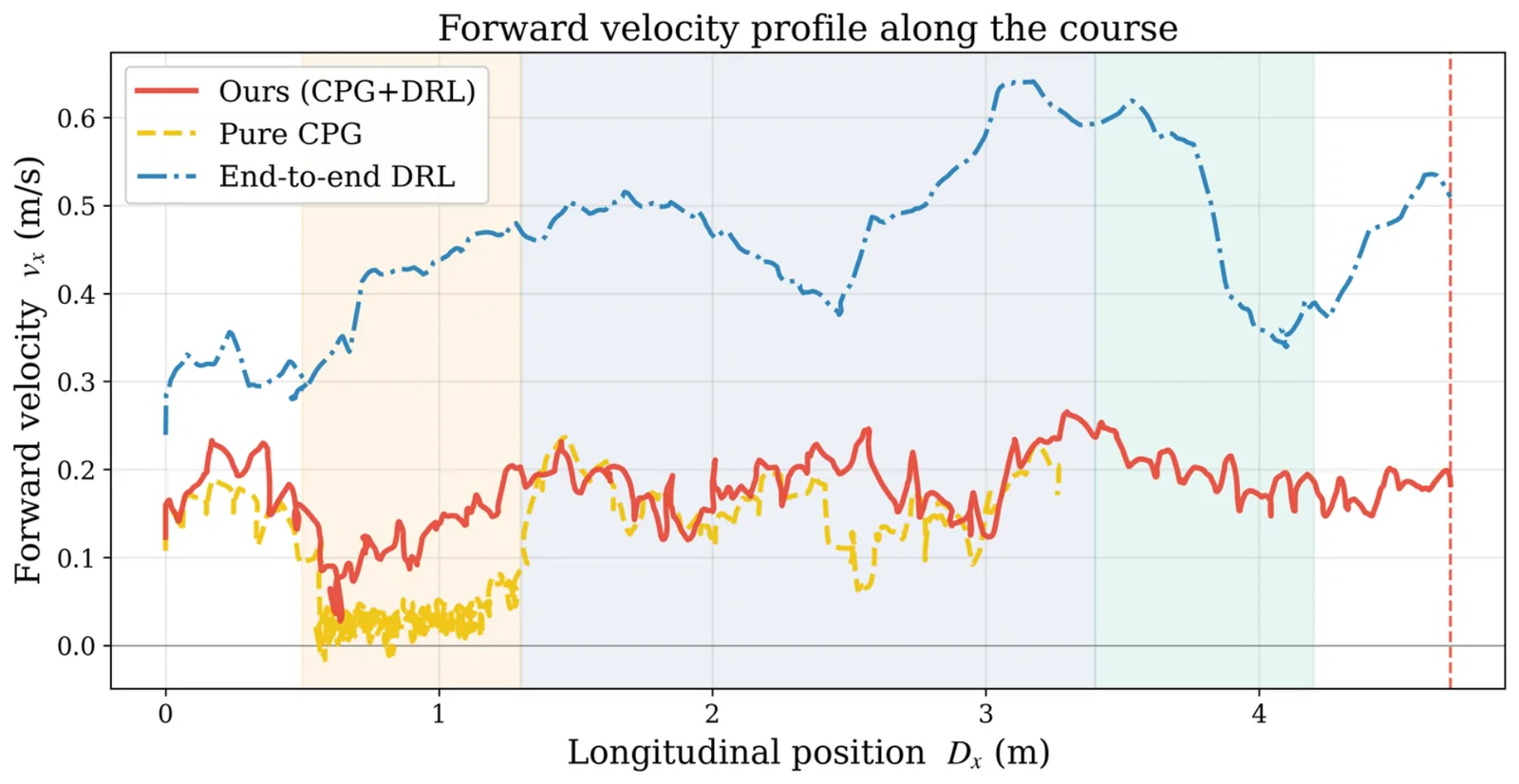
Forward velocity profiles along the terrain-transition course for the proposed method and the two baselines.

After the route-level trajectory was established, forward velocity was examined to evaluate propulsion continuity, three colors are used to correspond to uphill, irregular ground, and downhill, as shown in Figure 4. The proposed method maintained a moderate and relatively smooth velocity over the complete route and reached about 4.7 m. Pure CPG slowed to nearly zero through much of the early uphill interval and ended near 3.3 m. End-to-end DRL reached the endpoint but showed higher and more variable velocity, including a clear decrease around 3.9–4.1 m. Thus, the proposed method provided the most consistent propulsion profile rather than the highest instantaneous speed.

**Figure 5:**
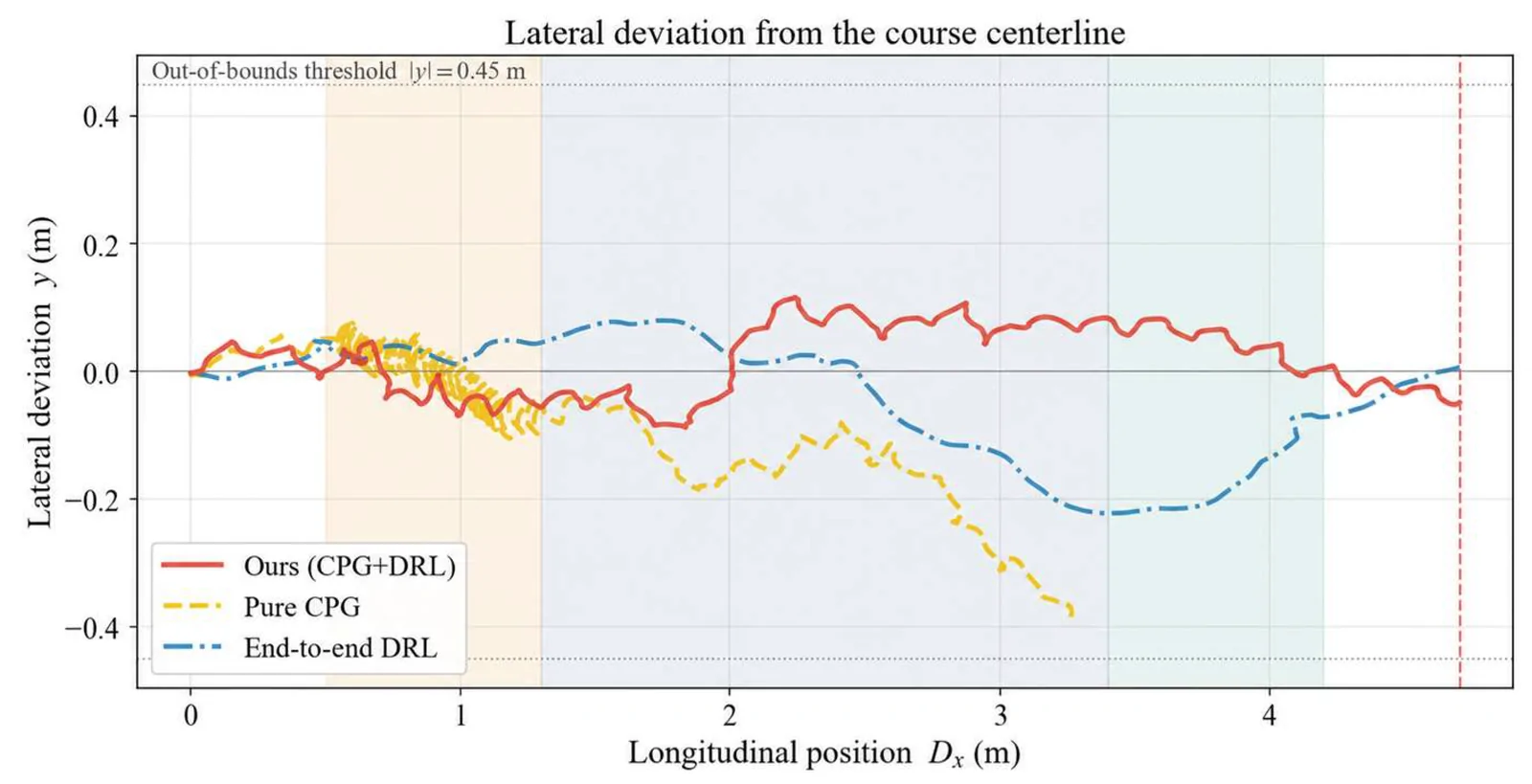
Lateral deviation from the course centerline during terrain-transition locomotion.

Because successful traversal also requires path keeping, the lateral deviation from the course centerline is reported in Figure 5. The proposed method remained well inside the |y| = 0.4 m boundary and was generally confined to approximately −0.1 to 0.12 m over the full route, ending close to the centerline. Pure CPG accumulated a progressively larger negative deviation and reached approximately −0.38 m before its profile ended near 3.3 m. End-to-end DRL also reached the endpoint in the representative profile, but developed a sustained negative offset of about −0.2 m through part of the irregular and downhill region before returning toward the centerline. Considering the magnitude and persistence of the deviation, the proposed method showed the most consistent lateral path keeping.

**Figure 6:**
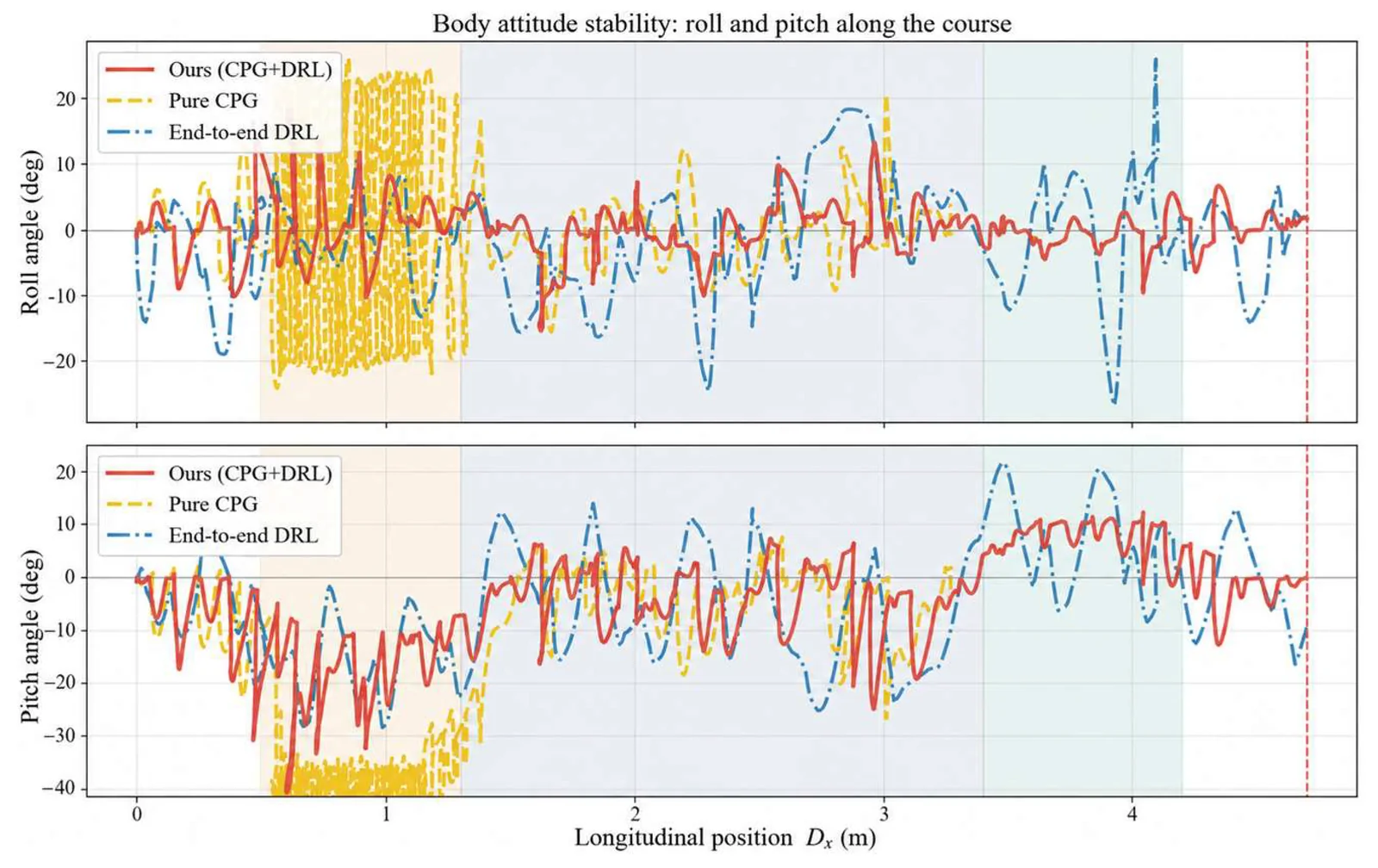
Roll and pitch attitude responses along the terrain-transition course.

To assess body stability during terrain transitions, Figure 6 compares roll and pitch responses. Pure CPG showed sustained large roll oscillations and pitch values near −40 degrees in the early transition interval. End-to-end DRL exhibited repeated large roll and pitch excursions throughout the route. The proposed method still showed transient pitch changes during slope transitions, but avoided sustained high-frequency roll oscillations, maintained a smaller overall attitude range, and generally recovered toward near-zero roll and pitch at the endpoint.

Overall, the simulation results show that fixed-parameter CPG can provide a basic rhythmic gait, but has limited adaptability to complex terrain. End-to-end DRL has a more expressive action space, but its training and execution stability were insufficient under the tested high-dimensional action formulation. By restricting the policy action to the three-dimensional CPG-parameter space, the proposed method preserved the phase-coordinated structure of the tripod gait while allowing PPO to adjust stride amplitude, swing-phase frequency, and steering from terrain-related proprioceptive feedback. This design improved route completion, velocity continuity, lateral stability, and body-attitude stability.

### 4.4. Simulation and Physical Traversal Validation

**Figure 7:**
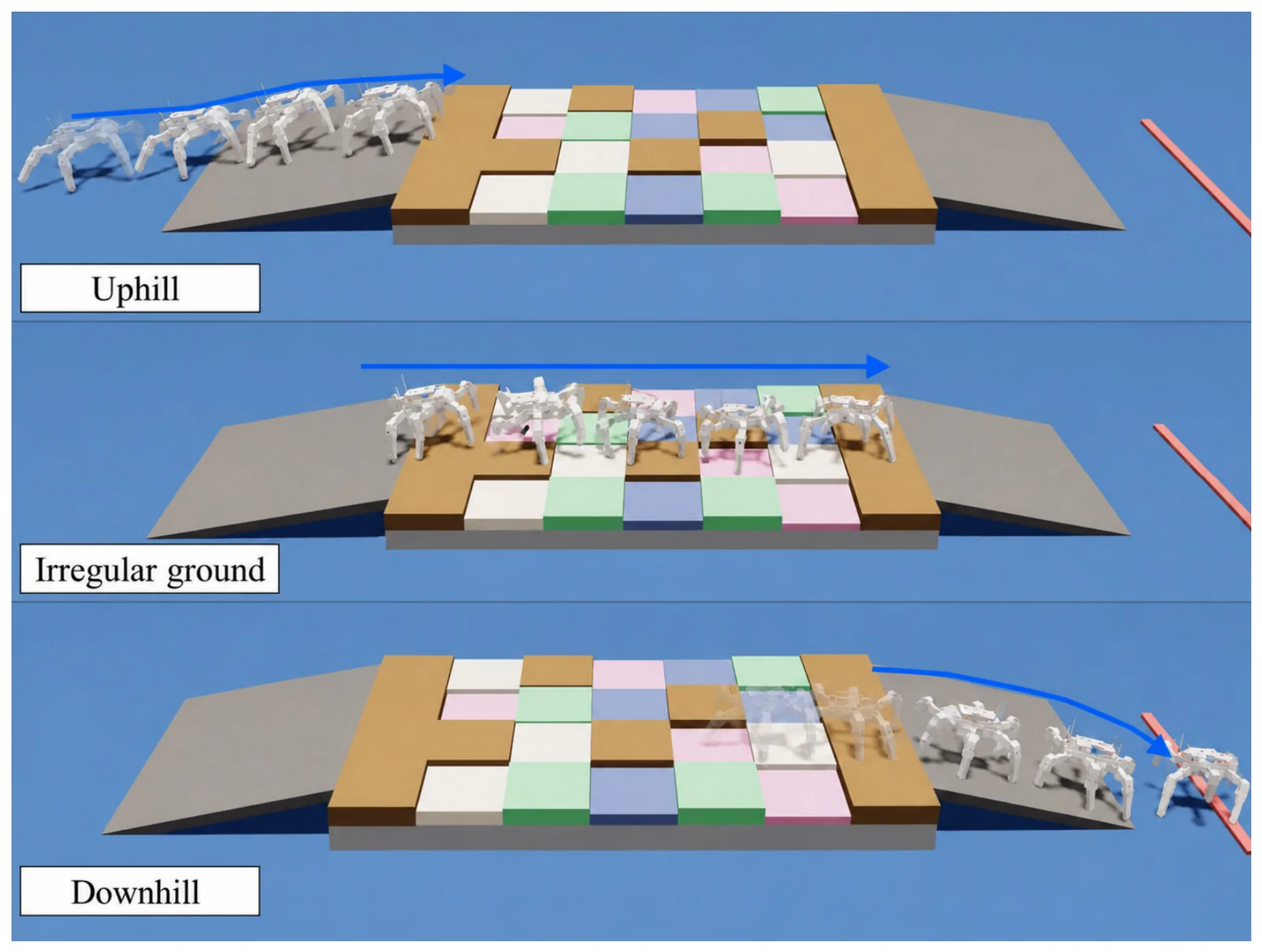
Simulated traversal sequence of the hexapod robot over continuous terrain transitions.

**Figure 8:**
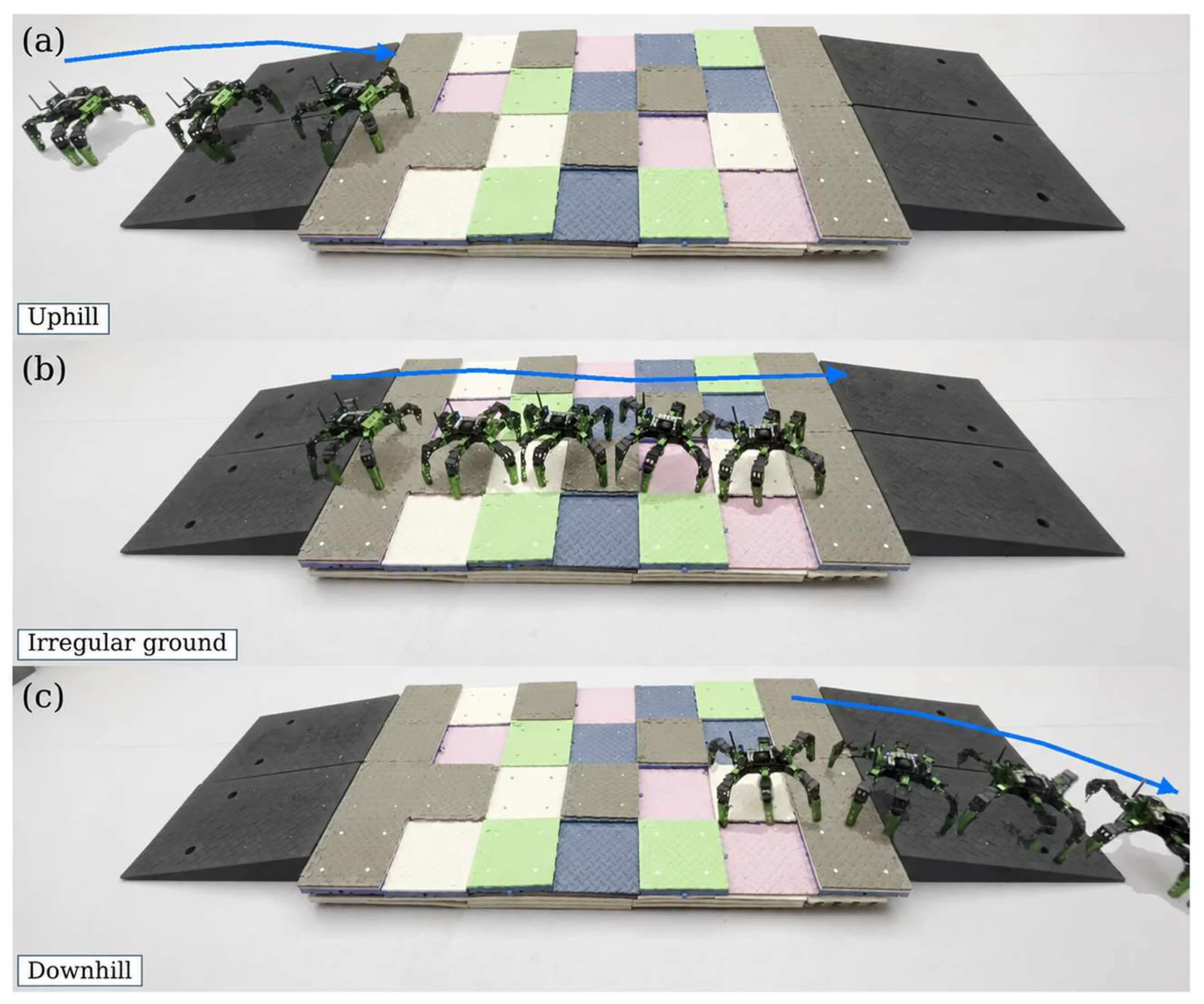
Physical traversal sequence of the JetHexa robot over continuous terrain transitions: (**a**) uphill-ramp traversal, (**b**) traversal over irregular raised blocks, and (**c**) downhill-ramp traversal. Within each panel, the representative robot poses are ordered chronologically from left to right, and the blue arrows indicate the direction of travel. Only selected keyframes are retained to reduce visual occlusion and improve readability.

To link the quantitative results with full-task execution, traversal sequences were recorded in simulation and on the physical platform, as shown in Figure 7 and Figure 8. The robot crossed uphill, irregular raised-terrain, and downhill sections in sequence. In both figures, the blue curves and arrows indicate forward traversal; in Figure 7, the red oblique line marks the terminal boundary rather than the motion direction. Without terrain-category input, terrain-specific controller switching, or policy reset, the robot completed the continuous transitions, demonstrating cross-terrain adaptation under the proposed CPG + DRL strategy.

In simulation, the robot maintained stable forward propulsion after entering the uphill section, without obvious attitude divergence or locomotion interruption. Over the irregular raised terrain, the trajectory showed local curvature and adjustment, indicating online gait-parameter regulation in response to foot-contact disturbances and body-state changes. During downhill traversal, the robot continued to move along the target direction and gradually recovered a smoother periodic rhythm after leaving the complex terrain. These simulated behaviors are illustrated in Figure 7 and were consistent with the trajectory, velocity, lateral deviation, and attitude-response results described above. The simulation policy was deployed on the physical robot without policy retraining or weight fine-tuning. The policy weights, 50 Hz control rate, and action bounds were retained, while fixed deployment adaptations were applied, including IMU filtering, CPG-based joint-state construction, torque-to-position command conversion, and fixed Coxa, swing, stride, and turn gains.

The corresponding physical traversal sequence in Figure 8 further verified the executability of the learned strategy. The blue arrows show the robot progressing from left to right through the uphill ramp, the irregular raised blocks, and the downhill ramp. The superimposed physical poses visually document continuous progression across the three terrain sections and a final position beyond the downhill segment. Compared with simulation, the real platform additionally involved foot–ground impact, nonuniform friction, and local deformation of the terrain blocks; nevertheless, the sequence shows completion of the continuous terrain-transition task without visible falling or stalling.

For the physical platform, ten archived IMU recordings were analyzed, yielding mean full-record roll and pitch RMS values of 12.95° and 14.42°, respectively. Because some recordings included post-trial manual handling and the logs lacked synchronized external position and trial-event annotations, these values are reported only as provisional attitude statistics, while quantitative success rate, velocity, and lateral deviation evaluation will require repeated experiments with external motion tracking.

Overall, the combined simulation and physical results showed that the proposed CPG + DRL framework enabled stable hexapod locomotion over continuously changing complex terrain without explicit terrain recognition or discrete gait switching. The CPG supplied a periodic rhythmic prior that preserved basic gait coordination, whereas DRL modulated rhythmic parameters online from proprioceptive states, allowing the robot to maintain locomotion continuity under slope changes, foot-contact disturbances, and locally discontinuous support. These results demonstrate that the parameterized learning-control framework not only improved task completion in simulation but also remained executable on the physical robot platform, indicating potential for cross-terrain generalization.

### 4.5. Comparison with Recent Technologies

To clarify the superiority of the proposed framework, Table 5 summarizes a performance-oriented comparison with recent learning-based hexapod locomotion technologies. Because recent studies use different robot platforms, terrain definitions, sensory configurations, and evaluation metrics, a fully standardized numerical benchmark is not yet available. Therefore, the comparison focuses on the reported task scope, control structure, performance evidence, and the specific advantage addressed by the present work, following the benchmarking perspective emphasized in recent DRL-based legged-locomotion reviews [43].

The comparison shows that the proposed framework is superior not because it optimizes a single steady-state metric, but because it combines three properties that are rarely evaluated together in recent hexapod studies: a unified controller, a three-dimensional rhythmic-parameter action space, and continuous cross-terrain validation. In the matched experiments of this study, these design choices improved long-distance success by 79 percentage points over Pure CPG and 68 percentage points over end-to-end DRL at 4.7 m, while preserving velocity continuity, lateral path keeping, and bounded body-attitude responses during terrain transitions.

## 5. Conclusions

This study presented a unified proprioception-driven DRL-CPG framework for adaptive hexapod locomotion across continuous terrain transitions. The framework used a CPG network to preserve rhythmic gait coordination and a PPO policy to map a 46-dimensional proprioceptive observation to a three-dimensional modulation action for amplitude, swing-phase frequency, and turn. Simulation results showed faster reward convergence, an approximately 420–430 late-stage episodic return, an 88% success rate at 4.7 m, comparatively smooth propulsion, limited lateral deviation, and recoverable roll/pitch responses relative to the fixed CPG and end-to-end DRL baselines. Compared with recent learning-based hexapod locomotion technologies, the proposed framework emphasizes unified proprioceptive control across sequential terrain transitions rather than isolated terrain adaptation or terrain-specific controller switching. The physical traversal sequence further showed that the learned parameter modulation could be executed on the JetHexa platform under contact and terrain uncertainties. These results indicate that parameterized learning over rhythmic gait primitives is a practical control strategy for blind hexapod locomotion across terrain transitions. Nevertheless, the current physical validation remains primarily qualitative, and repeated trials with synchronized external motion tracking are required for rigorous quantitative evaluation of success rate, velocity tracking, and lateral deviation.

## Figures and Tables

**Table 1:** Reward and penalty functions with corresponding coefficients for policy training.

Reward/Penalty Term	Reward/Penalty Function	Condition	Weight
$\mathrm{Linear}\;\mathrm{velocity}\;\mathrm{tracking}\;\mathrm{reward}\;r_{v}$	$\exp\left(-\frac{{\left\|v_{xy,t}^{\mathrm{cmd}}-v_{xy,t}\right\|}^{2}}{\sigma_{v}^{2}}\right)$	$\left|d_{y}\right|<0.45m$	15.0
$\mathrm{Yaw}\;\mathrm{angular}\;\mathrm{velocity}\;\mathrm{tracking}\;\mathrm{reward}\;r_{\omega }$	$\exp\left(-\frac{{\left(\omega_{z}^{\mathrm{cmd}}-\omega_{z}\right)}^{2}}{\sigma_{\omega }^{2}}\right)$	—	0.2
$\mathrm{Goal}-\mathrm{reaching}\;\mathrm{reward}\;r_{g}$	$I\left(d_{x}>x_{\mathrm{off}}\right)I\left(d_{y}<0.45m\right)$	$d_{x}>x_{\mathrm{off}},\;\left|d_{y}\right|<0.45m$	150.0
$\mathrm{Lateral}\;\mathrm{deviation}\;\mathrm{penalty}\;c_{l}$	$d_{y}^{2}$	—	−1.2
$\mathrm{Out}-\mathrm{of}-\mathrm{bounds}\;\mathrm{penalty}\;c_{b}$	$c_{b}=\left\{\begin{array}{ll} \rho^{2}, & \rho <1\\ \min\left(\rho^{3},5\right), & \rho \geq 1 \end{array}\right.$	$\rho =d_{y}/0.35m$	−2.5
$\mathrm{Heading}\;\mathrm{penalty}\;c_{h}$	${\left|\mathrm{wrap}\left(\psi -\psi_{ref}\right)\right|}^{2}$	Relative IMU yaw $\;\psi_{ref}=0$	−2.0
$\mathrm{Termination}\;\mathrm{penalty}\;c_{t}$	terminated	termination event	−20.0
$\mathrm{Motion}\;\mathrm{and}\;\mathrm{actuation}\;\mathrm{regularization}\;c_{s}$	${\left(v_{z}\right)}^{2}$ $,\;{\left\|\omega_{xy}\right\|}^{2}$ $,\;{\left\|\tau \right\|}^{2}$	—	−0.5, −0.05, −1 × 10^−5^

**Table 2:** PPO hyperparameters.

Hyperparameters	Value
Learning Rate	$1\times 10^{-4}$
Batch Size	6144
Number of Epochs	5
Gamma	0.99
GAE Lambda	0.95
Clip Range	0.2
Network Architecture	[256, 256]
Activation Function	ELU

**Table 3:** Success rates (%) at key forward distances for different methods.

Method	Distance (m)									
	1.3	1.6	1.9	2.2	2.5	2.8	3.1	3.4	4.2	4.7
Ours	100%	100%	100%	98%	98%	96%	94%	93%	91%	88%
Pure CPG	59%	54%	42%	36%	33%	27%	22%	17%	11%	9%
End-to-end DRL	95%	91%	75%	64%	54%	43%	36%	32%	22%	20%

Note: Each method was evaluated over 256 episodes (768 episodes in total). For a key distance d, an episode was counted as successful if its maximum forward displacement before the first termination reached or exceeded d. It was counted as a failure at d if lateral out-of-bounds termination (offset greater than 0.40 m from the initial centerline), excessive body inclination (approximately 1.0 rad), or the 60 s timeout occurred before d was reached. The reported percentages are therefore cumulative distance-arrival rates and are rounded to the nearest integer.

**Table 4:** Ablation results of the CPG modulation channels.

Method	Active Modulation Channels	Success Rate (%)	Evaluation Return (Mean ± SD)	Mean Distance (m)
Full CPG + DRL	*μ_t_*, *ω*_sw,_*_t_*, *δ_t_* (3D)	87.89	1306.04 ± 687.78	5.62
Without turn	*μ_t_*, *ω*_sw,_*_t_* (2D)	12.89	401.38 ± 528.29	3.20
Amplitude only	*μ_t_* (1D)	3.52	149.34 ± 298.53	1.78
Frequency only	*ω*_sw,_*_t_* (1D)	0.00	416.18 ± 71.40	3.74

**Table 5:** Comparison between recent hexapod locomotion technologies and present method.

Method Category	Key Feature	Main Limitation	Distinguishing Characteristic
Terrain-classification gait switching [8,10,11]	Terrain-dependent gait/policy selection	Requires reliable terrain labels	No terrain classification or switching
Fixed/rule-based CPG [7,15]	Predefined rhythmic coordination	Weak online adaptability	Online CPG parameter modulation
CPG-RL adaptation [16,19]	RL-enhanced rhythmic control	Limited emphasis on continuous transitions	Continuous terrain-transition evaluation
End-to-end DRL [39,40]	Joint-level learned control	High-dimensional exploration	Three-dimensional CPG action space
General legged RL adaptation [31,36]	Robust learned locomotion	Mostly non-hexapod or non-CPG-specific	Hexapod-specific DRL-CPG design
Proposed method	PPO-modulated CPG using proprioception	Depends on parameter bounds and transfer quality	Improved route completion and stability

## Data Availability

The data presented in this study are available from the corresponding author upon reasonable request. The simulation code, terrain models, trained policies, raw logs, and analysis scripts are not publicly available at this stage due to ongoing research and intellectual property considerations, but may be made available by the corresponding author under reasonable request and appropriate use conditions.

## Abbreviations

The following abbreviations are used in this manuscript:

CPG	Central pattern generator
DRL	Deep reinforcement learning
PPO	Proximal policy optimization
MDP	Markov decision process
PD	Proportional-derivative
GAE	Generalized advantage estimation
DoF	Degree of freedom
